# BiomedSQL: Text-to-SQL for Scientific Reasoning on Biomedical Knowledge Bases

**Published:** 2025-10-09

**Authors:** Mathew J. Koretsky, Maya Willey, Owen Bianchi, Chelsea X. Alvarado, Tanay Nayak, Nicole Kuznetsov, Adi Asija, Sungwon Kim, Mike A. Nalls, Daniel Khashabi, Faraz Faghri

**Affiliations:** 1 Center for Alzheimer’s Disease and Related Dementias, NIA, NIH;; 2 DataTecnica LLC;; 3 Johns Hopkins University;; 4 Laboratory of Neurogenetics, NIA, NIH

## Abstract

Biomedical researchers increasingly rely on large-scale structured databases for complex analytical tasks. However, current text-to-SQL systems often struggle to map qualitative scientific questions into executable SQL, particularly when implicit domain reasoning is required. We introduce *BiomedSQL*, the first benchmark explicitly designed to evaluate scientific reasoning in text-to-SQL generation over a real-world biomedical knowledge base. BiomedSQL comprises 68,000 question/SQL query/answer triples generated from templates and grounded in a harmonized BigQuery knowledge base that integrates gene–disease associations, causal inference from omics data, and drug approval records. Each question requires models to infer domain-specific criteria, such as genome-wide significance thresholds, effect directionality, or trial phase filtering, rather than rely on syntactic translation alone. We evaluate a range of open- and closed-source LLMs across prompting strategies and interaction paradigms. Our results reveal a substantial performance gap: GPT-o3-mini achieves 59.0% execution accuracy, while our custom multi-step agent, BMSQL, reaches 62.6%, both well below the expert baseline of 90.0%. BiomedSQL provides a new foundation for advancing text-to-SQL systems capable of supporting scientific discovery through robust reasoning over structured biomedical knowledge bases. Our dataset is publicly available at https://huggingface.co/datasets/NIH-CARD/BiomedSQL, and our code is open-source at https://github.com/NIH-CARD/biomedsql.

## 1 Introduction

Modern biomedical research is increasingly data-centric. Electronic health records (EHRs), high-throughput assays, and population-scale studies populate large databases that researchers query daily. Natural-language interfaces, particularly text-to-SQL systems, offer the promise of democratizing access to these resources. However, most current systems treat query generation as a syntactic translation task, mapping question structure to SQL templates without deeper domain understanding.

This abstraction breaks down in biomedical contexts. Domain experts routinely ask questions such as *“What SNPs are most significantly associated with Alzheimer’s disease?”* or *“What drugs target genes up-regulated in Parkinson’s disease?”*, questions grounded in implicit scientific conventions, such as statistical thresholds, drug approval pathways, and causal inference across multiple modalities.

These domain-specific conventions, e.g., genome-wide significance cutoffs, trial phase filtering, or effect-size interpretation, are invisible from the schema alone. While general-purpose text-to-SQL benchmarks (e.g., Spider (Yu et al., 2018), BIRD (Li et al., 2023)) have advanced the field, they do not evaluate the scientific reasoning required in complex domains. Similarly, EHR-focused benchmarks (Wang et al., 2020a; Lee et al., 2022) emphasize temporal logic or patient retrieval, but do not isolate or rigorously test **scientific reasoning on large-scale databases** that is required for interpreting biomedical data. This includes inferring statistical significance thresholds or chaining multi-step filtering logic across ontologies the way a skilled biomedical analyst would.

To address this gap, we introduce **BiomedSQL**, the first benchmark specifically designed to evaluate **scientific reasoning in SQL generation** within the biomedical domain. BiomedSQL contains 68,000 biomedical question/SQL query/answer triples that reflect realistic, complex scientific queries. These queries were templated from a set of 40 unique questions that were generated and verified by domain experts for their real-world biomedical research impact and ability to be answered through the use of structured data. These are executable against a harmonized, publicly available BigQuery database integrating gene–disease associations, multi-omic causal inferences, and drug approval records. These data sources were also verified as being the most up-to-date by domain experts that use them in their day-to-day computational research activities. Each question in BiomedSQL requires models to translate nuanced, qualitative scientific language into precise, executable SQL logic.

**Figure 1:**
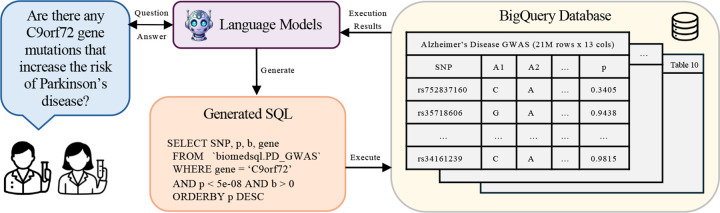
Example text-to-SQL workflow used to evaluate LLM performance on BiomedSQL. Given a question and the database schema information, an LLM must generate a SQL query and use its execution results to return a natural language response.

Figure 1 provides an overview of our evaluation pipeline, where we task various LLMs in a variety of prompting and interaction scenarios on their ability to generate accurate SQL queries and summarize the execution results into a concise, natural language response based on the provided question. Our evaluation on BiomedSQL indicates significant room for improvement in deploying LLMs on text-to-SQL workflows for biomedical knowledge bases. The top-performing model, GPT-o3-mini, only achieves an execution accuracy of 59.0%, even when provided with sample table rows, example queries, and explicit domain-specific instructions (e.g., significance thresholds). Our custom multi-step agent, BMSQL, improves this to 62.6%, but still trails expert performance (90%).

### Our contributions are as follows:

- **Benchmark:** We introduce BiomedSQL, a benchmark of 68,000 augmented question/SQL query/answer triples designed to evaluate scientific reasoning capabilities in text-to-SQL systems on a realistic, multi-table biomedical knowledge base.
- **Infrastructure:** We release a harmonized BigQuery database, expert-authored gold queries, execution results, and a toolkit for performance evaluation and agent orchestration.
- **Evaluation:** We assess a range of models, prompting styles, and multi-step interaction paradigms, including a custom-built text-to-SQL system (BMSQL), revealing a 30–40% gap compared to domain expert-level performance.

## 2 Related Work

We organize related work into four strands: (1) general-purpose text-to-SQL benchmarks, (2) text-to-SQL benchmarks for clinical databases, (3) evaluations of scientific reasoning in NLP, and (4) a direct comparison between BiomedSQL and previous benchmarks.

### General-purpose text-to-SQL benchmarks:

Text-to-SQL research has been largely driven by cross-domain benchmarks. Early work such as Seq2SQL (Zhong et al., 2017) introduced SQL generation for simple, single-table queries. Spider (Yu et al., 2018) expanded generalization challenges by spanning 200 multi-table databases, catalyzing the development of schema linking techniques. More recently, KaggleDBQA (Lee et al., 2021) and BIRD (Li et al., 2023) added realism by incorporating enterprise-scale data and requiring attention to data quality, joins, and latency. Despite progress, top models still underperform humans by 15–20% on execution accuracy for general-purpose benchmarks like BIRD. These benchmarks emphasize syntactic translation and schema generalization, not the domain-specific reasoning that underlies many queries in scientific disciplines. In contrast, BiomedSQL uses a multi-table biomedical schema enriched with domain-specific semantics, requiring models to interpret statistical conventions and biomedical ontologies—capabilities not previously assessed.

### Text-to-SQL benchmarks for clinical databases:

Several efforts have targeted clinical database querying for EHRs. MIMICSQL (Wang et al., 2020b) presented a synthetic SQL benchmark over MIMIC-III but was limited by its narrow schema. EHRSQL (Lee et al., 2022) advanced realism by crowdsourcing 1,000+ natural language queries from clinicians across MIMIC-III and eICU, highlighting challenges in temporal logic, answerability, and data sparsity. Recent datasets have diversified query paradigms across relational, document, and graph data models (Sivasubramaniam et al., 2024), reflecting the increasing heterogeneity of EHR data. These benchmarks focus on patient-centric information retrieval and assess the ability to map schema-dependent queries. Conversely, BiomedSQL targets scientific discovery in biomedical research, where queries require implicit scientific reasoning such as applying significance thresholds, combining multi-omic associations, or resolving drug–gene–disease relationships. This emphasis offers a complementary evaluation to clinical datasets by focusing on reasoning-heavy data exploration.

### Evaluating scientific reasoning in NLP:

Scientific reasoning has emerged as a critical frontier in NLP for tasks requiring multi-hop inference, evidence synthesis, and structured decision-making. Benchmarks such as SciFact (Wadden et al., 2020) and EntailmentBank (Dalvi et al., 2021) evaluate scientific claim verification and multi-step reasoning over textual evidence. Prompting techniques like Chain-of-Thought (Wei et al., 2022) and ReAct (Yao et al., 2023a) have demonstrated improved performance on multi-step reasoning tasks in both general and biomedical settings. More recent efforts have extended evaluation to structured data, such as TabularBench (Wang et al., 2025), Hierarchography (Gao et al., 2025), and SQL-RL (Ma et al., 2025), which assess LLM reasoning over tables, ontologies, and relational programs. Despite these advances, critical challenges remain in aligning model reasoning with biomedical standards of rigor, safety, and explainability. BiomedSQL addresses this gap by evaluating models on their ability to infer and operationalize scientific reasoning, including statistical thresholds, ontology resolution, and complex filtering, in text-to-SQL generation over large-scale biomedical knowledge bases.

### Benchmark comparison.

We compare BiomedSQL to several recent text-to-SQL benchmarks in Table 1. BiomedSQL is unique in four ways: (1) it contains the largest number of question/SQL/answer triples, (2) it features some of the longest average SQL queries (second only to EHRSQL), (3) it explicitly targets scientific reasoning rather than schema translation, and (4) it evaluates both the generated SQL and the model’s natural language response. BiomedSQL is also the only biomedical domain-specific benchmark that tests the model’s ability to utilize knowledge outside of the scope of the schema itself. Additionally, it is the only benchmark using BigQuery, a cloud-native SQL dialect, thus further simulating deployment-relevant environments.

## 3 BiomedSQL Construction

BiomedSQL is designed to evaluate scientific reasoning in text-to-SQL generation over structured biomedical knowledge. We construct it by: (1) harmonizing a multi-source relational database to support biomedical queries, (2) authoring gold-standard SQL annotations from domain experts, and (3) augmenting the dataset from templates to produce 68,000 question/SQL query/answer triples.

### 3.1 Relational Database Construction

We first constructed a relational database that spans ten core tables drawn from trusted biomedical resources, ensuring sufficient coverage for answering the full set of 68,000 questions. Data was pre-processed for consistency and deployed to BigQuery for efficient querying and public reproducibility.

Our primary data sources include the **OpenTargets Platform** (Targets, 2024), which aggregates gene–disease–drug associations, and **ChEMBL** (Méndez et al., 2024), a manually curated database of bioactive molecules and pharmacological data. OpenTargets data was retrieved via FTP and cleaned manually, while ChEMBL data was accessed through Google BigQuery and normalized by flattening nested fields. Together, these sources provide a unified schema of gene–disease links, drug–target pairs, trial status, and pharmacodynamics.

To support questions involving statistical genetics, we included summary statistics from large-scale GWAS studies of Alzheimer’s disease (Bellenguez et al., 2022) and Parkinson’s disease (Nalls et al., 2019), obtained from the GWAS Catalog. We retained SNP-level data including p-values, rsIDs, allele frequencies, and nearest-gene mappings after quality control filtering.

We integrated causal inference data from **omicSynth** (Alvarado et al., 2024), which applies summary-data-based Mendelian randomization (SMR) to identify multiomic biomarkers with putative causal links to neurodegenerative diseases. These datasets enable reasoning over associations not directly stated but statistically inferred, e.g. *“What metabolites causally influence Parkinson’s progression?”*

All tables were normalized and uploaded in Parquet format to Google Cloud BigQuery. A full schema and column listing are provided in Appendix A.1. To support future expansions, we have curated additional tables that extend BiomedSQL’s coverage to broader omics and clinical-trial data.

### 3.2 SQL Annotation and Augmentation

#### Gold SQL authoring.

To ensure executable grounding, a domain expert manually wrote gold-standard SQL queries for each of the 40 seed questions drawn from CARDBiomedBench (Bianchi et al., 2025). Each query was crafted to retrieve the minimum evidence necessary to answer the question, avoiding SELECT * patterns and capping results at 100 rows. Two additional analysts reviewed all queries for syntactic correctness and semantic fidelity.

#### Programmatic scaling.

Each of the 40 queries was then templated and automatically expanded using entity substitution. We aligned these templates to the full set of 68,000 QA pairs in CARDBiomedBench by programmatically inserting disease, gene, SNP, and compound mentions into the query templates. All generated SQL queries were executed on the BigQuery database to obtain execution results, which serve as ground-truth evidence for evaluating LLM-generated SQL queries.

This pipeline produced a benchmark where each QA pair is linked to an executable SQL query and its result. This enables precise evaluation of models’ ability to translate scientific questions into domain-grounded, semantically valid, and executable SQL logic.

## 4 Dataset Analysis

### Task distribution and SQL complexity.

**Figure 2:**
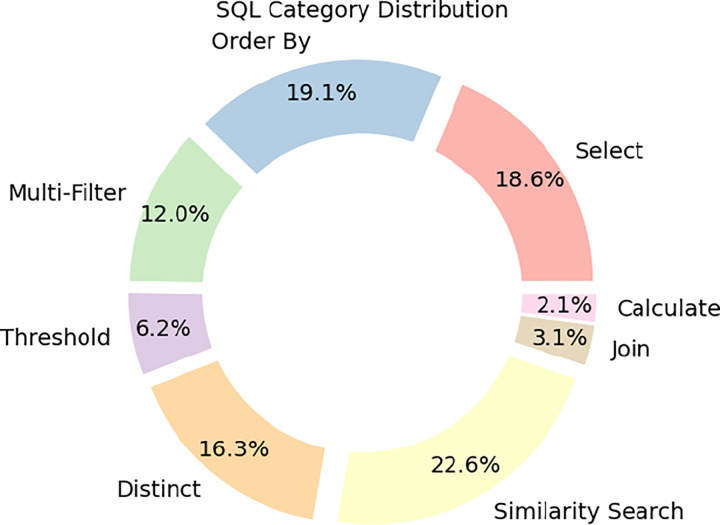
SQL category distribution.

To characterize the scientific and computational complexity of BiomedSQL, we annotated all 68,000 question–query pairs with SQL operation types and biomedical reasoning categories. Table 2 defines the SQL operation categories, and Figure 2 shows their empirical distribution. Simpler operations—such as *Select*, *Order-By*, and *Calculate*—require relatively shallow syntactic parsing, which LLMs tend to perform well on. In contrast, operations such as *Multi-Filter*, *Threshold*, *Join*, and *Similarity Search* present greater difficulty, as they demand multi-step logical composition, implicit schema linking, or pattern-based retrieval.

### Scientific reasoning categories.

To probe scientific reasoning, we classified BiomedSQL queries into three reasoning categories reflecting cognitive processes typical of biomedical experts:

1. **Operationalizing implicit scientific conventions:** Queries often invoke domain-specific concepts (e.g., “significantly associated SNPs”) that imply non-obvious statistical thresholds, such as *p* < 5 × 10^−8^ for GWAS hits or directionality based on beta coefficients. These conventions are rarely explicit in schemas and must be inferred by models.
2. **Incorporating missing contextual knowledge:** Experts often incorporate auxiliary data (e.g., drug approval status or clinical trial phase) even when not directly mentioned. For instance, determining if a drug is “approved” for a condition requires disambiguating indication-specific trial phase information beyond any binary “approved” flag.
3. **Executing complex multi-hop reasoning workflows:** Many questions in BiomedSQL require chaining relational operations across multiple tables. For example, “Which tissues are genes associated with Parkinson’s disease most significantly expressed in?” requires a four-step inference over gene–disease, gene–expression, tissue annotations, and statistical ranking. LLMs often struggle to translate such multi-hop logic into valid, executable SQL.

Additional biological reasoning categories and visualization of their distribution are provided in Appendix A.2 (Table 6, Figure 4).

## 5 Experiments

The goal of our experiments is to assess how well LLMs can translate biomedical natural language questions from BiomedSQL into accurate and executable BigQuery SQL queries. We evaluate models across different interaction paradigms and prompting configurations, comparing both SQL execution accuracy and the quality of the natural language answers they generate.

### 5.1 Experimental Setup

#### Models.

We evaluate a range of state-of-the-art open-source and proprietary LLMs. Open-source models include **LLaMA-3.1** (70B, 405B) and **Qwen-2.5-Coder** (14B, 32B). Closed-source models include the **GPT** family (GPT-4o, GPT-4o-mini, GPT-o3-mini), the **Gemini-2.0-flash** family (flash and flash-lite), and **Claude-3.7-sonnet**. This selection spans a diverse range of parameter scales, computational cost profiles, and architectural design philosophies.

#### Isolated SQL generation.

We first assess models in a single-turn text-to-SQL setting. Each model receives a baseline prompt containing: (1) the natural language biomedical question from the benchmark, (2) the database schema describing tables, columns, and relationships, and (3) simple instructions on how to structure the SQL query and remain consistent with the database schema.

To study prompt sensitivity, we vary prompt structure along several axes: (1) adding sample table rows (*3-rows*, *5-rows*), (2) adding few-shot examples (*1-shot*, *3-shot*, *5-shot*), (3) adding explicit domain-specific instructions (e.g., statistical thresholds via *stat-instruct*), and (4) a combined variant that includes *3-rows*, *3-shot*, and *stat-instruct* (*combo*). Prompt templates are provided in Appendix A.3.

#### Interaction paradigms.

Beyond single-turn prompts, we investigate multi-step paradigms that allow iterative reasoning and query refinement. These systems can request schema details, propose intermediate queries, and update their approach before presenting a final SQL query. These paradigms were evaluated using GPT and Gemini models. We experiment with four architectural variants:

1. **ReAct:** A prompt-orchestrated approach where schema validation, syntax checking, and other external tools are invoked within multi-step SQL generation steps (Yao et al., 2023b). The react prompts used are detailed in Appendix A.4.
2. **Schema Indexing:** Schema descriptions are dynamically retrieved using LlamaIndex to support contextual grounding and table selection.
3. **DAIL-SQL:** We adapt DAIL-SQL (Gao et al., 2023) for use on BiomedSQL. DAIL-SQL is a state-of-the-art text-to-SQL solution that retireves relevant example SQL queries based on the question and injects them into the prompt for more accurate query generation. It is consistently near the top of leaderboards for popular benchmarks like Spider and BIRD.
4. **Multi-step query refinement:** We implement an iterative text-to-SQL architecture, called BMSQL, where an initial query is refined through feedback loops based on intermediate results or execution errors, emulating expert-level refinement processes. The implementation details of BMSQL are shown in Appendix A.5.

#### SQL execution metrics.

We report three SQL performance metrics: **Execution Accuracy (EX)**, **Jaccard Index (JAC)**, and **Syntax Error Rate (SER)**. Execution Accuracy is a widely used text-to-SQL metric (Yu et al., 2018; Li et al., 2023) which represents the proportion of questions in the evaluation set for which the LLM-generated query and the ground-truth query return identical results. We adapt EX for our use case to measure row-wise set equality, comparing the set of UUIDs returned in the case of SELECT * queries or the set of numeric values returned in the case of COUNT and other calculation queries. Jaccard Index (Costa, 2021), or intersection over union, is a metric for gauging the similarity of two sets. It tells us how close the LLM-generated SQL query results are to the ground-truth. Unlike EX, JAC will still credit a query that returns slightly more or less rows than the ground-truth, making it a more lenient metric. Finally, Syntax Error Rate is simply the proportion of questions in the evaluation set for which the LLM-generated SQL query was not executable.

#### BioScore.

To evaluate natural language responses, we adopt BioScore (Bianchi et al., 2025), an LLM-as-a-judge metric computed using GPT-4o. BioScore includes:

- **Response Quality Rate (RQR):** Proportion of factually correct answers. Measures how often a model provides correct answers.
- **Safety Rate (SR):** Proportion of abstentions among all incorrect or abstained answers. Assesses a model’s ability to abstain from answering when uncertain.

All metric definitions and prompts are provided in Appendix A.6. A correlation analysis between the SQL execution and BioScore metrics is presented in Appendix A.7. To mitigate concern over the use of LLM-as-a-judge metrics, a correlation analysis between LLM-generated and domain expert-generated BioScores is presented in Appendix A.8.

#### Domain Expert Baseline.

Two expert biomedical analysts answered a quiz over a representative sample of questions. For each, they generated SQL queries, execution results, and natural language answers. We report mean EX, JAC, and RQR. SR and SER are not available for this format, as experts could not abstain and produced valid SQL in all cases.

### 5.2 Evaluation Results

#### LLMs struggle with scientific reasoning in SQL generation.

Table 3 shows that even top-performing models such as GPT-o3-mini (EX = 53.5%, JAC = 60.4%, RQR = 73.3%) fall short of domain expert performance (90–95%). GPT-4o performs slightly worse (EX = 46.9%, JAC = 54.7%, RQR = 71.2%). Among open-weight models, despite their small size, Qwen-2.5-Coder-32B achieves competitive EX (40.8%) and Qwen-14B attains strong RQR (62.1%), outperforming Llama models that dwarf them in terms of parameters. Claude-3.7-sonnet exhibits the best SR (43.0%), indicating better abstention behavior.

#### Prompt variations provide small gains.

Table 8 in Appendix A.9 shows that the *combo* prompt yields the best improvement for GPT-o3-mini (ΔEX=+5.5%, ΔJAC=+5.7%, ΔRQR=+4.5%). However, these gains come at nearly 3 times the token usage, suggesting limited cost-effectiveness. Passing raw table rows alone showed negligible benefit, underscoring that schema-level understanding matters more than content memorization.

#### Interaction paradigms yield mixed results.

Table 4 shows that schema indexing underperforms in both EX and RQR, likely due to its use of simple table descriptions and lightweight grounding. However, it exhibits the best SR (e.g., Index-GPT-4o = 66.9%), indicating it effectively abstains when uncertain. ReAct marginally improves EX for GPT variants but does not perform consistently across models. This suggests that ReAct-style prompts may need to be tuned to optimize performance on different models. Also, its high token usage makes it less practical for deployment. DAIL-SQL shows strong performance, rivaling that of BMSQL on all three models tested. However, it is important to note that even this state-of-the-art text-to-SQL approach still trails expert-level performance by 30%.

#### BMSQL is the strongest performer overall.

Our custom system, BMSQL, outperforms all baselines. GPT-o3-mini with BMSQL achieves 62.6% EX and 69.2% JAC—both best in class. Paired with Gemini, BMSQL reaches 84.6% RQR, rivaling even domain experts on answer quality. However, execution accuracy remains significantly lower than expert benchmarks. These results highlight the value of domain-specific multi-step agents in structured biomedical tasks.

## 6 Analysis

To better understand model behavior beyond aggregate metrics, we analyze performance across SQL task types and evaluate the effects of increased inference-time compute.

### Performance across SQL categories.

**Figure 3:**
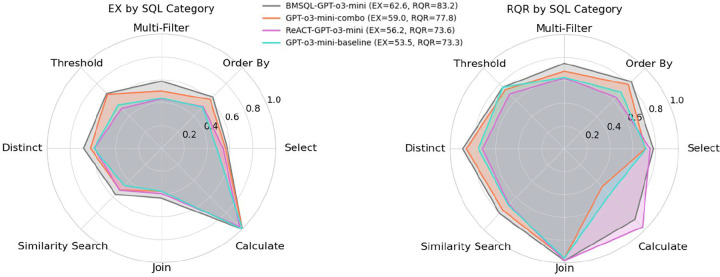
Distribution of performance across SQL categories for GPT-o3-mini in terms of EX (left) and RQR (right), across four prompting and interaction paradigms.

Figure 3 presents radar plots showing the distribution of Execution Accuracy (EX) and Response Quality Rate (RQR) across SQL categories, as defined in §4. We evaluate GPT-o3-mini across four settings: (1) baseline prompt, (2) *combo* prompt, (3) ReAct prompting, and (4) BMSQL.

For EX, performance across SQL categories remains relatively stable across prompting strategies. As anticipated, models struggle most with *Join*, *Similarity Search*, and *Multi-Filter* queries, which require multi-table reasoning, implicit filtering, or fuzzy matching. Surprisingly, *Select* queries also show mid-range performance; however, this category includes a large portion of questions in BiomedSQL, so mean-level performance is expected. For RQR, BMSQL exhibits the most balanced performance across categories, likely due to its ability to: (1) apply domain-specific instructions (e.g., p-value thresholds, trial status), (2) compare thresholded vs. unthresholded results, (3) refine queries via execution feedback. This reinforces the benefits of multi-step pipelines in biomedical reasoning.

### Common SQL errors.

In order to determine the kinds of mistakes the LLMs are making in generating the SQL queries, we defined the following five error categories: (1) incorrect tables, (2) missing threshold, (3) incorrect threshold, (4) incorrect aggregations, and (5) syntax error. More precise definitions of each error category are presented in Appendix A.10, along with the counts of each error type for six of the top-performing models in Table 9.

Incorrect table selection is the most common error in the LLM-generated SQL queries, followed by missing or incorrect application of statistical thresholds. These results speak to the high correlation between the tables of the benchmark database and the lack of biomedical domain-specific reasoning ability by frontier LLMs, even when correct statistical thresholds are provided directly to the model.

### Effect of inference-time compute.

We next evaluate how model performance changes when given the opportunity to reflect and revise outputs over multiple reasoning steps. Specifically, we allow BMSQL-GPT-o3-mini to examine its initial SQL query, execution result, and answer, then choose to perform up to two additional passes if the outputs appear insufficient.

Results in Table 5 show that increasing inference steps yields marginal gains. From 1-pass to 3-pass, EX remains flat (62.6% → 61.7%), while RQR increases slightly (83.1% → 85.5%). Improvements in SR and elimination of syntax errors (SER = 0.0%) suggest that most corrections involve fixing syntax or abstaining rather than reformulating the query logic. Notably, BMSQL rarely chooses to invoke a third pass, as reflected in the minor token increase between the 2-pass and 3-pass settings.

### Effect of database size.

BiomedSQL was created from a database of ten tables. While the count is small compared to other benchmarks, the size of the tables is much larger, spanning up to 72 million rows and 30 columns. This presents the LLMs with the difficult task of finding the correct columns to use and applying the proper thresholds based on the semantics of the presented question. The smaller table count is also justified by model performance on the benchmark: even the top performers trail expert-level execution accuracy by nearly 30%.

To thoroughly elucidate model performance on BiomedSQL, we added ten new tables to our database, including additional GWAS results from different diseases, drug mechanism of action data, and population-level allele frequencies. We evaluate GPT-o3-mini across three settings on this larger schema: (1) baseline prompt, (2) *combo* prompt, and (3) BMSQL. Table 10 in Appendix A.11 shows the results from this analysis. For the baseline and combo experiments, the drop in performance for EX, JAC, and RQR when moving to the larger schema ranges from 7–10%. The performance drop seen by BMSQL is less dramatic, ranging from 2–4% depending on the metric. These experiments show that, as expected, adding more tables to the database increases the difficulty of the text-to-SQL generation task for state-of-the-art models. We also discuss future benchmark expansion plans in §7.

## 7 Discussion and Limitations

### Use of template questions.

BiomedSQL was constructed from a set of 40 template questions. While templating can cause homogeneous samples compared to a real-world setting, it is common in the text-to-SQL community (Gao et al., 2023; Gan et al., 2021; Saparina and Lapata, 2024). Popular text-to-SQL benchmarks that use a large set of templates, or don’t use templates at all, often crowdsource the generation of text-to-SQL tasks. For example, BIRD (Li et al., 2023) crowdsourced its questions from domains that are known by the general population. They only require a person to have some familiarity with the topic and with SQL syntax (sports, movies, sales, etc.). Conversely, BiomedSQL requires domain experts to generate valid questions and SQL tasks that are valuable to the biomedical research landscape. These experts are advanced biomedical data scientists that implement complex analysis workflows in their day-to-day. Therefore, the generation of these tasks cannot be crowdsourced, requiring the use of templates. We also cite the difficulty of the benchmark task despite our use of templates, as top models still trail expert performance by nearly 30%.

### Multiple valid SQL solutions.

While gold SQL queries in BiomedSQL were authored by domain experts and independently verified by analysts, they do not represent the only correct way to retrieve relevant data for a given question. Biomedical questions often permit multiple semantically valid formulations, e.g., using alternative joins, filters, or aggregations. To account for this, we evaluate models using a combination of metrics, including execution-based (EX, JAC) and LLM-judged response quality (RQR), to more robustly reflect real-world answer utility.

### Use of BigQuery SQL.

While we recognize that reliance on a cloud-specific dialect such as BigQuery may limit direct comparability with prior work, we view this as an important design decision. Cloud-native SQL dialects are increasingly common in production systems, especially in biomedical informatics pipelines. Evaluating LLMs in this setting brings new challenges, including vendor-specific functions, syntax, and query planning, that have been underexplored in the research community.

### Future directions.

We plan to expand our set of template questions to cover the experimental 20-table schema. We also have additional tables covering extensive GWAS and CRISPR screen data that would also be a valuable addition to the benchmark. We plan to convert the benchmark to SQLite for easier integration with more general-purpose text to SQL systems such as DIN-SQL (Pourreza and Rafiei, 2023) and CHESS (Talaei et al., 2024). Finally, a public-facing leaderboard would facilitate researcher attempts to saturate the biomedical reasoning text-to-SQL task.

## 8 Conclusion

We present BiomedSQL, the first large-scale text-to-SQL benchmark explicitly designed to evaluate scientific reasoning during SQL generation in the biomedical domain. Our experiments show that BiomedSQL poses a substantial challenge to state-of-the-art LLMs, with execution accuracy and answer quality still lagging far behind domain expert performance.

By focusing on implicit domain conventions, multi-step reasoning, and structured biomedical data, BiomedSQL highlights key limitations of current systems and offers a rigorous testbed for future research. We believe this benchmark is a critical step toward building more capable, trustworthy text-to-SQL systems that can broaden access to biomedical knowledge and accelerate discovery for researchers across disciplines.

## 9 Reproducibility Statement

Our self-contained code to reproduce all of the main experimental results from this paper is publicly available at https://github.com/NIH-CARD/biomedsql. The benchmark dataset, as well as the tabular data that comprises our BigQuery database, is also publicly available at https://huggingface.co/datasets/NIH-CARD/BiomedSQL. In addition, Appendix A.13 details the expected compute resources needed for all experiments. Upon publication, we plan to make our BigQuery database publicly available in order to support reproducible research.

## Supplementary Material

1

## Figures and Tables

**Table 1:** Comparison of BiomedSQL to other text-to-SQL benchmarks. BiomedSQL uniquely evaluates scientific reasoning and natural language responses while supporting BigQuery execution.

Dataset	Number of Questions	Number of Queries	Avg. Tokens	Knowledge	Template	Scientific Reasoning	NL Response	BigQuery
MIMICSQL (Wang et al., 2020b)	10,000	10,000	57.4	✗	✓	✗	✗	✗
EHRSQL (Lee et al., 2022)	24,000	24,000	**109.9**	✗	✓	✗	✗	✗
SM3 (Sivasubramaniam et al., 2024)	10,000	40,000	26.1	✗	✓	✗	✗	✗
BIRD (Zhang et al., 2023)	12,751	12,751	50.6	✓	✗	✗	✗	✗
**BiomedSQL**	**68,227**	**68,227**	96.4	✓	✓	✓	✓	✓

**Table 2:** Description of SQL query categories in BiomedSQL.

SQL Category	Description
Select	Retrieves specific columns from one or more tables.
Distinct	Retrieves unique values from specified columns.
Join	Combines rows across multiple tables via relational keys.
Multi-Filter	Applies compound filtering logic (AND, OR, NOT).
Threshold	Filters based on logical or statistical thresholds (e.g., p-values).
Calculate	Performs arithmetic operations (e.g., counts, averages).
Order-By	Sorts the result set by specified columns.
Similarity Search	Performs pattern-based retrieval using LIKE, regex, or full-text search.

**Table 3:** State-of-the-art LLMs struggle with scientific reasoning-based text-to-SQL tasks (*Domain expert baselines not available for SR, SER, and token count as described in §5.1).

Model	EX (%) ↑	JAC (%) ↑	RQR (%)↑	SR (%) ↑	SER (%) ↓	# Tokens
Domain Expert	90.0	90.0	95.0	NA*	NA*	NA*
GPT-4o	46.9 _(±4.2)_	54.7 _(±3.8)_	71.2 _(±3.8)_	26.1 _(±3.7)_	1.3 _(±0.9)_	3,689
GPT-4o-mini	35.9 _(±4.0)_	41.4 _(±3.9)_	60.6 _(±4.1)_	23.3 _(±3.5)_	11.0 _(±2.6)_	89,612
GPT-o3-mini	**53.5**_**(±4.2)**_	**60.4**_**(±3.8)**_	**73.3**_**(±3.7)**_	29.4 _(±3.8)_	**0.2**_**(±0.4)**_	3,942
Gemini-2.0-flash	33.7 _(±4.0)_	37.0 _(±3.9)_	71.1 _(±3.8)_	27.2 _(±3.7)_	4.2 _(±1.7)_	3,692
Gemini-2.0-flash-lite	17.9 _(±3.2)_	18.1 _(±3.2)_	41.0 _(±4.1)_	26.4 _(±3.7)_	8.4 _(±2.3)_	3,280
Claude-3.7-sonnet	45.4 _(±4.2)_	49.8 _(±4.0)_	69.8 _(±3.8)_	**43.0**_**(±4.1)**_	1.6 _(±1.1)_	3,805
Qwen-2.5-Coder-14B	37.0 _(±4.0)_	32.4 _(±3.9)_	62.1 _(±4.1)_	42.5 _(±4.1)_	11.0 _(±2.6)_	3,453
Qwen-2.5-Coder-32B	40.8 _(±4.1)_	44.4 _(±4.0)_	58.2 _(±4.1)_	61.0 _(±4.1)_	15.7 _(±3.1)_	3,612
Llama-3.1-70B	34.4 _(±4.0)_	39.8 _(±3.9)_	57.0 _(±4.1)_	37.0 _(±4.0)_	6.0 _(±2.0)_	3,547
Llama-3.1-405B	38.1 _(±4.1)_	42.5 _(±4.0)_	57.9 _(±4.1)_	41.7 _(±4.1)_	4.6 _(±1.7)_	3,456

**Table 4:** Complex interaction paradigms provide mixed performance (*Gemini-2.0-flash is the Gemini model used for these experiments).

Model	EX (%) ↑	JAC (%) ↑	RQR (%)↑	SR (%) ↑	SER (%) ↓	# Tokens
ReAct-GPT-4o	49.6 _(±4.2)_	57.9 _(±3.8)_	67.2 _(±3.9)_	8.9 _(±2.4)_	0.0 _(±0.0)_	14,286
ReAct-GPT-o3-mini	56.2 _(±4.2)_	64.8 _(±3.6)_	73.6 _(±3.7)_	13.2 _(±2.8)_	0.0 _(±0.0)_	13,317
ReAct-Gemini*	48.9 _(±4.2)_	56.6 _(±3.8)_	60.4 _(±4.1)_	10.2 _(±2.5)_	**0.0**_**(±0.0)**_	13,205
Index-GPT-4o	25.5 _(±3.6)_	28.3 _(±3.6)_	44.1 _(±4.2)_	**66.9**_**(±3.9)**_	27.5 _(±3.7)_	1,110
Index-GPT-o3-mini	27.1 _(±3.7)_	30.6 _(±3.7)_	44.1 _(±4.1)_	47.5 _(±4.2)_	2.0 _(±0.1)_	1,899
Index-Gemini*	46.1 _(±4.2)_	48.5 _(±4.1)_	54.2 _(±4.2)_	59.6 _(±4.1)_	8.1 _(±2.3)_	787
DAIL-SQL-GPT-4o	54.8 _(±4.2)_	58.1 _(±3.4)_	75.5 _(±3.4)_	63.4 _(±4.0)_	6.6 _(±2.1)_	1,624
DAIL-SQL-GPT-o3-mini	61.2 _(±4.1)_	63.6 _(±4.0)_	81.4 _(±3.3)_	42.1 _(±4.1)_	0.0 _(±0.0)_	1,318
DAIL-SQL-Gemini*	53.1 _(±4.2)_	58.8 _(±3.4)_	82.8 _(±3.1)_	30.6 _(±3.7)_	0.0 _(±0.0)_	1,185
BMSQL-GPT-4o	60.4 _(±4.1)_	67.2 _(±3.6)_	79.8 _(±3.4)_	64.5 _(±4.0)_	4.9 _(±1.8)_	32,819
BMSQL-GPT-o3-mini	**62.6**_**(±4.1)**_	**69.2**_**(±3.6)**_	83.1 _(±3.1)_	38.0 _(±4.1)_	2.6 _(±1.2)_	39,470
BMSQL-Gemini*	55.9 _(±4.2)_	61.3 _(±3.9)_	**84.6**_**(±3.0)**_	32.1 _(±3.9)_	0.2 _(±0.4)_	22,045

**Table 5:** Increased inference time compute has little effect on performance of BMSQL-GPT-o3-mini.

Model	EX (%) ↑	JAC (%) ↑	RQR (%) ↑	SR (%) ↑	SER (%) ↓	# Tokens
1-pass	**62.6**_**(±4.1)**_	69.2 _(±3.6)_	83.1 _(±3.1)_	**38.0**_**(±4.1)**_	2.6 _(±1.2)_	39,470
2-pass	62.1 _(±4.1)_	**69.4**_**(±3.5)**_	84.2 _(±3.1)_	29.1 _(±3.8)_	**0.0**_**(±0.0)**_	53,773
3-pass	61.7 _(±4.1)_	69.2 _(±3.5)_	**85.5**_**(±2.9)**_	36.7 _(±4.0)_	0.0 _(±0.0)_	55,948
