# Supplementary material for: BiomedSQL: Text-to-SQL for Scientific Reasoning on Biomedical Knowledge Bases
Source: ArXiv. 2025 Oct 9:arXiv:2505.20321v3. Preprint. [Version 3] (PMC12478439)
Supplement: 1 [file NIHPP2505.20321V3-supplement-1.pdf]

A Appendix

A.1 Database tables.

This section provides schema details and a short description of the ten core tables in the BiomedSQL BigQuery database. Database Tables 1-2, 6-8, and 9-10 are generated from sources that are under the CC0 1.0 License or are otherwise designated to the public domain. Database Tables 3, 5 and Database Table 4 are generated from sources under the CC BY 4.0 and CC BY-NC 4.0 License, respectively.

Database Table 1: Alzheimer's Disease GWAS (21.1M rows, 13 columns).

```
Table: AlzheimerDisease_CombinedGeneData_UUID
Description: Summary statistics from the largest publicly available GWAS of Alzheimer's Disease in a
European population (Bellenguez et al., 2022).
Schema:
  - Name: UUID | Type: STRING | Mode: REQUIRED
  - Name: SNP | Type: STRING | Mode: NULLABLE
  - Name: A1 | Type: STRING | Mode: NULLABLE
  - Name: A2 | Type: STRING | Mode: NULLABLE
  - Name: freq | Type: FLOAT | Mode: NULLABLE
  - Name: b | Type: FLOAT | Mode: NULLABLE
  - Name: se | Type: FLOAT | Mode: NULLABLE
  - Name: p | Type: FLOAT | Mode: NULLABLE
  - Name: chr_37 | Type: INTEGER | Mode: NULLABLE
  - Name: bp_37 | Type: INTEGER | Mode: NULLABLE
  - Name: chr_38 | Type: INTEGER | Mode: NULLABLE
  - Name: bp_38 | Type: INTEGER | Mode: NULLABLE
  - Name: nearestGene | Type: STRING | Mode: NULLABLE
```

Database Table 2: Parkinson's Disease GWAS (7.8M rows, 13 columns).

```
Table: ParkinsonDisease_CompleteGeneData_No23andMe
Description: Summary statistics from the largest publicly available GWAS of Parkinson's Disease in a
European population (Nalls et al., 2019).
Schema:
  - Name: UUID | Type: STRING | Mode: REQUIRED
  - Name: SNP | Type: STRING | Mode: NULLABLE
  - Name: A1 | Type: STRING | Mode: NULLABLE
  - Name: A2 | Type: STRING | Mode: NULLABLE
  - Name: freq | Type: FLOAT | Mode: NULLABLE
  - Name: b | Type: FLOAT | Mode: NULLABLE
  - Name: se | Type: FLOAT | Mode: NULLABLE
  - Name: p | Type: FLOAT | Mode: NULLABLE
  - Name: chr_37 | Type: INTEGER | Mode: NULLABLE
  - Name: bp_37 | Type: INTEGER | Mode: NULLABLE
  - Name: chr_38 | Type: INTEGER | Mode: NULLABLE
  - Name: bp_38 | Type: INTEGER | Mode: NULLABLE
  - Name: nearestGene | Type: STRING | Mode: NULLABLE
```

Database Table 3: Alzheimer's Disease Gene Pathway Associations (542 rows, 5 columns).

```
Table: AlzheimerDisease_GeneAssoc_Pathways_UUID
Description: Summary statistics from a pathway-level analysis of gene sets in Alzheimer's Disease
(Zhang et al., 2021).
Schema:
  - Name: UUID | Type: STRING | Mode: REQUIRED
  - Name: genes | Type: STRING | Mode: NULLABLE
  - Name: size | Type: INTEGER | Mode: NULLABLE
  - Name: statistic | Type: FLOAT | Mode: NULLABLE
  - Name: p | Type: FLOAT | Mode: NULLABLE
```

Database Table 4: Parkinson's Disease Gene Pathway Associations (1,016 rows, 5 columns).

```
Table: ParkinsonDisease_GeneAssoc_Pathways_UUID
Description: Summary statistics from a pathway-level analysis of gene sets in Parkinson's Disease
(Elango et al., 2023).
Schema:
  - Name: UUID | Type: STRING | Mode: REQUIRED
  - Name: genes | Type: STRING | Mode: NULLABLE
  - Name: size | Type: INTEGER | Mode: NULLABLE
  - Name: statistic | Type: FLOAT | Mode: NULLABLE
  - Name: p | Type: FLOAT | Mode: NULLABLE
```

Database Table 5: Neurodegenerative Disease SMR Associations (1.7M rows, 31 columns).

```
Table: NeurodegenerativeDiseases_SMR_Genes_Full
Description: SMR results providing functional inferences between genetic variants and six neurodegenerative
diseases (Alvarado et al., 2024).
Schema:
  - Name: UUID | Type: STRING | Mode: REQUIRED
  - Name: Omic | Type: STRING | Mode: NULLABLE
  - Name: Disease | Type: STRING | Mode: NULLABLE
  - Name: probeID | Type: STRING | Mode: NULLABLE
  - Name: ProbeChr | Type: INTEGER | Mode: NULLABLE
  - Name: Gene | Type: STRING | Mode: NULLABLE
  - Name: Probe_bp | Type: INTEGER | Mode: NULLABLE
  - Name: topSNP | Type: STRING | Mode: NULLABLE
  - Name: topSNP_chr | Type: INTEGER | Mode: NULLABLE
  - Name: topSNP_bp | Type: INTEGER | Mode: NULLABLE
  - Name: A1 | Type: STRING | Mode: NULLABLE
  - Name: A2 | Type: STRING | Mode: NULLABLE
  - Name: Freq | Type: FLOAT | Mode: NULLABLE
  - Name: b_GWAS | Type: FLOAT | Mode: NULLABLE
  - Name: se_GWAS | Type: FLOAT | Mode: NULLABLE
  - Name: p_GWAS | Type: FLOAT | Mode: NULLABLE
  - Name: b_eQTL | Type: FLOAT | Mode: NULLABLE
  - Name: se_eQTL | Type: FLOAT | Mode: NULLABLE
  - Name: p_eQTL | Type: FLOAT | Mode: NULLABLE
  - Name: b_SMR | Type: FLOAT | Mode: NULLABLE
  - Name: se_SMR | Type: FLOAT | Mode: NULLABLE
  - Name: p_SMR | Type: FLOAT | Mode: NULLABLE
  - Name: p_SMR_multi | Type: FLOAT | Mode: NULLABLE
  - Name: p_HEIDI | Type: FLOAT | Mode: NULLABLE
  - Name: nsnp_HEIDI | Type: FLOAT | Mode: NULLABLE
  - Name: topRSID | Type: STRING | Mode: NULLABLE
  - Name: Omic_type | Type: STRING | Mode: NULLABLE
  - Name: Omic_tissue | Type: STRING | Mode: NULLABLE
  - Name: Disease_name | Type: STRING | Mode: NULLABLE
  - Name: Source | Type: STRING | Mode: NULLABLE
  - Name: func_sig | Type: STRING | Mode: NULLABLE
```

Database Table 6: Neurodegenerative Disease Allele Frequencies (72.2M rows, 6 columns).

```
Table: NeurodegenerativeDisease_AlleleFrequencies_UUID
Description: Allele frequencies from a cohort not containing Alzheimer's or Parkinson's disease cases
(Bergstrom et al., 2020).
Schema:
  - Name: UUID | Type: STRING | Mode: REQUIRED
  - Name: chr_38 | Type: INTEGER | Mode: NULLABLE
  - Name: SNP | Type: STRING | Mode: NULLABLE
  - Name: A1 | Type: STRING | Mode: NULLABLE
  - Name: A2 | Type: STRING | Mode: NULLABLE
  - Name: freq | Type: FLOAT | Mode: NULLABLE
```

Database Table 7: Drug Gene Targets (6,391 rows, 20 columns).

```
Table: DrugGeneTargets_ComprehensiveAnnotations_updated
Description: Details drug-gene relationships and offers a comprehensive view of drug development pipelines
(OpenTargets and ChEMBL).
Schema:
  - Name: UUID | Type: STRING | Mode: REQUIRED
  - Name: chemblIdentifier | Type: STRING | Mode: NULLABLE
  - Name: blackBoxWarning | Type: BOOLEAN | Mode: NULLABLE
  - Name: drugName | Type: STRING | Mode: NULLABLE
  - Name: drugMolecularType | Type: STRING | Mode: NULLABLE
  - Name: yearOfFirstApproval | Type: INTEGER | Mode: NULLABLE
  - Name: maxClinicalTrialPhase | Type: INTEGER | Mode: NULLABLE
  - Name: drugHasBeenWithdrawn | Type: BOOLEAN | Mode: NULLABLE
  - Name: drugIsApproved | Type: BOOLEAN | Mode: NULLABLE
  - Name: tradeNames_string | Type: STRING | Mode: NULLABLE
  - Name: drugSynonyms_string | Type: STRING | Mode: NULLABLE
  - Name: linkedDiseasesDrug_string | Type: STRING | Mode: NULLABLE
  - Name: linkedDiseasesCount | Type: INTEGER | Mode: NULLABLE
  - Name: newLinkedTargets_string | Type: STRING | Mode: NULLABLE
  - Name: numberLinkedTargets | Type: INTEGER | Mode: NULLABLE
  - Name: drugDescription | Type: STRING | Mode: NULLABLE
  - Name: drugActionType | Type: STRING | Mode: NULLABLE
  - Name: drugMechanismOfAction | Type: STRING | Mode: NULLABLE
  - Name: tradename_count | Type: INTEGER | Mode: NULLABLE
  - Name: synonyms_count | Type: INTEGER | Mode: NULLABLE
```

Database Table 8: Drug Target Indications (1.2M rows, 23 columns).

```
Table: DrugTargets_IndicationsAndTherapeuticUses
Description: Links drugs to specific indications, facilitating disease- and target-specific
therapeutic explorations (OpenTargets and ChEMBL).
Schema:
  - Name: UUID | Type: STRING | Mode: REQUIRED
  - Name: chemblId | Type: STRING | Mode: NULLABLE
  - Name: drugName | Type: STRING | Mode: NULLABLE
  - Name: tradeName | Type: STRING | Mode: NULLABLE
  - Name: drugType | Type: STRING | Mode: NULLABLE
  - Name: actionType | Type: STRING | Mode: NULLABLE
  - Name: targetType | Type: STRING | Mode: NULLABLE
  - Name: target | Type: STRING | Mode: NULLABLE
  - Name: approvedSymbol | Type: STRING | Mode: NULLABLE
  - Name: approvedName | Type: STRING | Mode: NULLABLE
  - Name: yearOfFirstApproval | Type: INTEGER | Mode: NULLABLE
  - Name: usan_year | Type: FLOAT | Mode: NULLABLE
  - Name: patent_no | Type: STRING | Mode: NULLABLE
  - Name: max_phase_for_ind | Type: FLOAT | Mode: NULLABLE
  - Name: mesh_id | Type: STRING | Mode: NULLABLE
  - Name: mesh_heading | Type: STRING | Mode: NULLABLE
  - Name: efo_id | Type: STRING | Mode: NULLABLE
  - Name: efo_term | Type: STRING | Mode: NULLABLE
  - Name: tradeNames_list | Type: STRING | Mode: NULLABLE
  - Name: tradename_count | Type: INTEGER | Mode: NULLABLE
  - Name: syns_list | Type: STRING | Mode: NULLABLE
  - Name: synonyms_count | Type: INTEGER | Mode: NULLABLE
  - Name: ct | Type: STRING | Mode: NULLABLE
```

Database Table 9: Drug Licensing (2,097 rows, 16 columns).

```
Table: DrugTargets_LiscensingAndUses
Description: Licensing, pharmaceutical company, and dosage information for specific drugs (FDA Purple Book).
Schema:
  - Name: UUID | Type: STRING | Mode: REQUIRED
  - Name: applicant | Type: STRING | Mode: NULLABLE
  - Name: blaNumber | Type: INTEGER | Mode: NULLABLE
  - Name: tradeName | Type: STRING | Mode: NULLABLE
  - Name: drugName | Type: STRING | Mode: NULLABLE
  - Name: blaType | Type: STRING | Mode: NULLABLE
  - Name: strength | Type: STRING | Mode: NULLABLE
  - Name: dosageForm | Type: STRING | Mode: NULLABLE
  - Name: routeOfAdministration | Type: STRING | Mode: NULLABLE
  - Name: productPresentation | Type: STRING | Mode: NULLABLE
  - Name: marketingStatus | Type: STRING | Mode: NULLABLE
  - Name: licensure | Type: STRING | Mode: NULLABLE
  - Name: submissionType | Type: STRING | Mode: NULLABLE
  - Name: licenseNumber | Type: INTEGER | Mode: NULLABLE
  - Name: productNumber | Type: INTEGER | Mode: NULLABLE
  - Name: center | Type: STRING | Mode: NULLABLE
```

Database Table 10: Drug Dosages (211k rows, 11 columns).

```
Table: DrugTargets_UsesAndDosages
Description: Dosage, route of administration, and strength information for specific drugs
(National Drug Code).
Schema:
  - Name: UUID | Type: STRING | Mode: REQUIRED
  - Name: productType | Type: STRING | Mode: NULLABLE
  - Name: tradeName | Type: STRING | Mode: NULLABLE
  - Name: drugName | Type: STRING | Mode: NULLABLE
  - Name: dosageForm | Type: STRING | Mode: NULLABLE
  - Name: dosageRoute | Type: STRING | Mode: NULLABLE
  - Name: labelerName | Type: STRING | Mode: NULLABLE
  - Name: activeDosage_strength | Type: STRING | Mode: NULLABLE
  - Name: activeIngredient_strength | Type: STRING | Mode: NULLABLE
  - Name: mechanismOfAction_pharma | Type: STRING | Mode: NULLABLE
  - Name: packageDescription | Type: STRING | Mode: NULLABLE
```

A.2 Biological reasoning categories.

Table 6 defines the biological reasoning categories that BiomedSQL challenges and Figure 4 shows their distribution among the set of 68,000 queries. The *GWAS Significance*, *SMR Significance*, and *Functional Significance* categories test the ability of LLMs to operationalize domain-specific statistical significance thresholds. Categories such as *Approval Status*, *Genetic Target*, and *Trial Phase* task LLMs with understanding and applying information about clinical trial phases for specific drugs and indications to generate a correct SQL query.

Table 6: Description of Biological Reasoning Categories in BiomedSQL.

**Bio Category**	**Description**
Approval Status	Retrieves information on the FDA approval status of a drug or indication.
Trial Phase	Retrieves information on the clinical trial phase a drug has reached.
GWAS Significance	Identifies variants that are GWAS significant for a disease ($p < 5e\text{-}08$).
SMR Significance	Identifies variants that are SMR significant for a disease ($p < 2.95e\text{-}06$).
Functional Significance	Identifies variants that are significant in a particular tissue for a disease.
Effect	Retrieves the effect size and direction for specific variants.
Genetic Target	Retrieves information on the genetic target of a drug.
Allele Frequency	Calculates allele frequencies for a variant or set of variants.
Metadata	Retrieves general information on a drug or genetic variant.

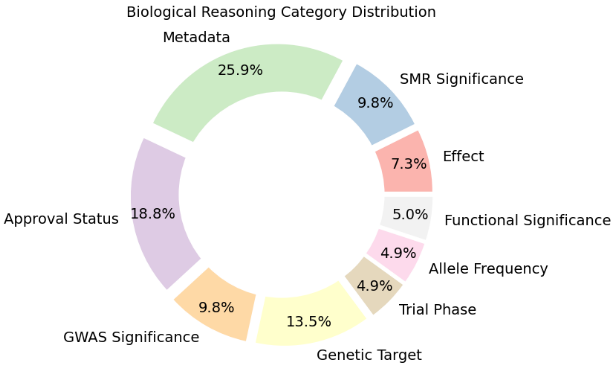

Figure 4: Distribution of biological reasoning query types.

A.3 Isolated SQL generation prompt templates.

This section provides details about the prompt engineering approaches used for the isolated SQL query generation experiments.

Prompt 1 contains the baseline prompt template that was used. For these experiments, the *db_schema* variable is replaced with the schema that is detailed in Appendix A.1. In the case of the *3-rows*, *5-rows*, and *combo* experiments, *db_schema* is replaced with a schema that includes the corresponding number of example rows for each table in the database.

Prompt 2 shows the example queries that were appended to the baseline prompt for use in the *1-shot*, *3-shot*, *5-shot*, and *combo* experiments. Note that these example queries stay the same regardless of the question from BiomedSQL being passed.

Prompt 3 details the statistical thresholding instructions that were appended to the baseline prompt for use in the *stat-instruct* and *combo* experiments.

Finally, Prompt 4 contains the prompt template that was passed to the LLMs for the generation of a final natural language response based on the question, generated SQL query, and execution results. This prompt was used throughout the isolated SQL generation experiments.

```
You are a data analyst and SQL developer experienced with biomedical data in Google BigQuery.
Your task is to translate the user's natural language question into a syntactically correct
Google BigQuery SQL query.

User's Natural Language Question:
{question}

Database Schema:
{db_schema}

Use these guidelines when generating the query:
    1. Review the database schema.
    2. Review the user's question.
    3. Generate a valid Google BigQuery SQL query that answers the question based on the schema.
    4. Always enclose table references in backticks, e.g. `project.dataset.table`.
    5. Make use of BigQuery-specific functions and syntax where appropriate
    (e.g., DISTINCT, aliases, ORDER BY).
    6. Always include the UUID column in your SELECT statements, except in cases of questions where the
    COUNT and ORDER BY functions are needed.
    7. Unless the user explicitly requests a different LIMIT, default your queries to LIMIT 100.
    8. Output ONLY the raw SQL query (no additional commentary or explanations).
    9. Avoid SELECT *; select only the necessary columns to answer the user's query.
    10. Ensure that any disease names that contain an apostrophe in the query are surrounded by
    double quotes (e.g., "Alzheimer's Disease").

Please only return the SQL query in the following format:
```
{{sql_query}}
```
```

Prompt 1: Baseline prompt template for the isolated SQL generation experiments.

```
Below are example BigQuery queries to guide you (for reference only, do not repeat verbatim unless needed
by the user's request):
Example 1:
    SELECT DISTINCT drugName, drugIsApproved, newLinkedTargets_string
    FROM `card-ai-389220.bio_sql_benchmark.DrugGeneTargets_ComprehensiveAnnotations_updated`
    WHERE newLinkedTargets_string LIKE "%TUBB %"
    AND drugIsApproved = TRUE
    LIMIT 1000;

Example 2:
    SELECT SNP, A1 AS effect_allele, freq AS effect_allele_frequency,
    A2 AS non_effect_allele, 1 - freq AS non_effect_allele_frequency
    FROM `card-ai-389220.bio_sql_benchmark.AlzheimerDisease_CombinedGeneData_UUID`
    WHERE SNP = 'rs61769339'
    LIMIT 10;

Example 3:
    SELECT drugName, newLinkedTargets_string, drugIsApproved
    FROM `card-ai-389220.bio_sql_benchmark.DrugGeneTargets_ComprehensiveAnnotations_updated`
    WHERE newLinkedTargets_string LIKE '%ACACA%'
    AND drugIsApproved = TRUE
    LIMIT 1000;

Example 4:
    SELECT topRSID, Disease, Gene, p_SMR_multi, p_HEIDI, b_SMR
    FROM `card-ai-389220.bio_sql_benchmark.NeurodegenerativeDiseases_SMR_Genes_Full`
    WHERE Disease = 'FTD' AND Gene = 'ORC3' AND p_SMR_multi < 2.95e-6
    LIMIT 100

Example 5:
    SELECT DISTINCT drugName, tradeNames_list, drugType, actionType, target, approvedSymbol, approvedName,
    yearOfFirstApproval, max_phase_for_ind, mesh_heading, efo_term
    FROM `card-ai-389220.bio_sql_benchmark.DrugTargets_IndicationsAndTherapeuticUses`
    WHERE (LOWER(efo_term) = "acute hepatic porphyria"
    OR LOWER(mesh_heading) = "acute hepatic porphyria") AND LOWER(drugType) = "oligonucleotide"
    AND yearOfFirstApproval > 0 AND max_phase_for_ind = 4.0
    LIMIT 100
```

Prompt 2: Example queries appended to the baseline prompt for the *n-shot* and *combo* experiments.

```
Use the following p-value thresholds for questions about statistical significance:
    1. p < 5e-8 for genome-wide significance.
    2. p_SMR < 2.95e-6 for SMR significance.
    3. p_SMR < 2.95e-6, p_HEIDI > 0.01 for functional significance.
```

Prompt 3: Statistical thresholding instructions appended to the baseline prompt for the *stat-instruct* and *combo* experiments.

```
You are a data analyst and SQL developer experienced with biomedical data in Google BigQuery.
Given the following question, SQL query, and SQL query execution results, please provide a concise answer.
Please do not use any information outside of the SQL query and SQL query execution results in your answer.
Question:
{question}
SQL Query:
{sql_query}
Execution Results:
{execution_results}
```

Prompt 4: Natural language response prompt template for the isolated SQL generation experiments.

A.4 REACT PROMPT TEMPLATE.

Prompt 5 shows the ReAct-style prompt template used in the interaction paradigm experiments. Similar to the baseline prompt *db_schema* is replaced with the schema that is detailed in Appendix A.1. *history_str* is replaced by the reasoning trace from previous steps that the LLM chooses to take. We allow the LLM to perform up to 5 iterations within the ReAct loop before a final answer is generated.

````
You are an expert SQL agent that uses step-by-step reasoning to answer questions about data in a
BigQuery database.

IMPORTANT: The dataset name is "{dataset_name}". Always qualify table names with this dataset name.
Example: SELECT * FROM {dataset_name}.table_name

Follow these steps:
    1. Think about how to translate the question into a SQL query.
    2. Decide which tables and columns are needed.
    3. Write a SQL query with explanatory comments.
    4. Verify the query syntax before executing.
    5. If the query has errors, fix them and try again.
    6. Once the query is successful, explain the results clearly.
    7. Always include the UUID column in your SELECT statements, except in cases of questions where
    the COUNT and ORDER BY functions are needed.
    8. Unless the user explicitly requests a different LIMIT, default your queries to LIMIT 100.
    9. Avoid SELECT *; select only the necessary columns to answer the user's query.
    10. Ensure that any disease names that contain an apostrophe in the query are surrounded by
    double quotes (e.g., "Alzheimer's Disease").

Your output MUST be a JSON object with these fields:
{{
    "thought": "Your reasoning about how to answer the question",
    "action": "One of 'verify_sql', 'execute_sql', or 'final_answer'",
    "action_input": "For verify_sql/execute_sql: the SQL query;
                     For final_answer: explanation of the results"
}}

IMPORTANT:
    - Your response must include valid JSON that can be parsed.
    - Do not include any explanations outside the JSON object.
    - Always qualify table names with the dataset name "{dataset_name}."

Make sure your SQL queries follow BigQuery SQL syntax and include helpful inline comments.

Question: {question}

Database Schema:
```
{db_schema}
```

Reasoning History:
{history_str}

Continue the reasoning process with the next step:
````

Prompt 5: ReAct prompt template for the interaction paradigm experiments.

A.5 BMSQL PROMPT TEMPLATES.

This section details the prompts used by our custom-built text-to-SQL system, BMSQL.

Prompt 6 provides the template for the first step in the BMSQL pipeline, which is using the schema to identify relevant tables and columns to generate a SQL query given the question. Once relevant columns are selected, Prompt 7 is used for BMSQL to generate a first attempt at a general SQL query that corresponds to the question. If the execution of this query fails, Prompt 8 is used to generate a query that resolves any syntax errors present in the original query. BMSQL is given up to three retries to correct any syntax errors at this step.

Prompt 9 is used to generate a query that applies any statistical thresholding rules that may be necessary to answer the question. If no statistical thresholding is needed, the general query is returned once again. Using the execution results from both the general and refined query, BMSQL is asked to generate a final response to the question given the instructions in Prompt 10.

Finally, Prompt 11 is used in the inference time compute experiments to give BMSQL an opportunity to deem the final response as insufficient to answer the question and take subsequent passes through the pipeline. On these subsequent passes, BMSQL tends to correct any syntax errors but rarely makes structural changes to the generated SQL queries.

The multi-stage query generation that BMSQL uses was designed to reflect how a domain expert might query a biomedical knowledge base by first checking if data is available for a given query, and applying statistical thresholding on a subsequent query. As seen throughout §5.2, BMSQL is a top performer in terms of both execution metrics and response quality.

```
You are a BioMedical Domain Expert with deep database knowledge. You have the following database schema:
{db_schema}

The user has asked a question about this biomedical data:
"{question}"

Your task:
    1. Identify the single table or multiple tables (if absolutely necessary) that would provide the
    *full* answer to this question.
    2. From these table(s), list *all columns* that might be relevant to fully answer the question.
    (Because a downstream aggregator will handle details, do NOT omit columns that may be relevant.)

Format your response **strictly** as:
TABLE_NAME: col1, col2, col3, ...
    - Provide no extra commentary or text.
    - If multiple tables are truly needed, list each in a new line, in the same format.
```

Prompt 6: BMSQL prompt template for selecting relevant columns.

```
You are a highly proficient BigQuery SQL generator in the biomedical domain.

Database schema:
{db_schema}

The user asked:
"{question}"

Previously identified relevant columns/tables:{relevant_columns}

Instructions:
    - Generate exactly one valid BigQuery SQL query that retrieves all relevant columns
    from the relevant_columns list.
    - Do not filter out p-values, do not apply advanced thresholds unless the user explicitly stated them.
    - If the user mentions FDA approval, include those columns.
    - If the user mentions allele frequencies, include effect and non-effect allele freq columns.
    - FROM clause: `{project_id}.{dataset_name}.table_name`
    - Always include the UUID column in your SELECT statements, except in cases of questions where
    the COUNT and ORDER BY functions are needed.
    - Unless the user explicitly requests a different LIMIT, default your queries to LIMIT 100.

Return only the final SQL in a markdown code block:
```sql
{{sql_query}}
```
```

Prompt 7: BMSQL prompt template for generating a first attempt general SQL query.

```
You are a SQL debugging assistant for Google BigQuery. Below is the database schema,
the failed query, and the error message or unexpected results:

=== DATABASE SCHEMA START ===
{db_schema}
=== DATABASE SCHEMA END ===

=== FAILED SQL QUERY START ===
```sql
{general_query}
```
=== FAILED SQL QUERY END ===

=== ERROR OR RESULTS START ===
{general_results}
=== ERROR OR RESULTS END ===

The user originally asked:
"{question}"

Relevant columns identified for answering this question:
{relevant_columns}

Your task:
    - Analyze the failed query and the error or result details.
    - Generate a corrected SQL query that resolves the issue,
    ensuring it's correct for BigQuery and fits the schema.

Format the corrected query as a valid SQL query in a markdown fenced block:
```sql
{{sql_query}}
```
```

Prompt 8: BMSQL prompt template for correcting a failed first attempt general SQL query.

```
You are a skillful BigQuery SQL refiner. The user might want additional thresholds or
see if there's advanced filtering needed, e.g. p-values or FDA approvals.

Original question: "{question}"

The previously generated SQL query was:
```sql
{sql_query}
```

The query's results (showing up to 10 rows):
{resp_str}

Database schema:
{db_schema}

Known threshold rules:
{threshold_rules}

If no extra thresholds or filters are implied, keep the same query.
Otherwise, produce a refined SQL with the new filters,
returning it in a markdown code block:
```sql
{{sql_query}}
```
```

Prompt 9: BMSQL prompt template for generating a refined SQL query that applies thresholding rules if necessary.

```
You are a BioMedical Domain expert that is returning a concise answer to the user's question
based on two sets of SQL queries and results. If not sure, say you do not know.

Question: {question}

SQL query 1: {sql_query_1}
Result 1: {result_1}

SQL query 2: {sql_query_2}
Result 2: {result_2}
```

Prompt 10: BMSQL prompt template for generating a natural language response to the question.

```
You are a biomedical domain and BigQuery expert that is determining if a text-to-SQL workflow
should be run again.
Based on the question SQL queries, their execution results, and the final answer,
determine if you are confident in the answer.

Use the following guidelines:
    1. If the SQL queries or answer contain errors, deem the answer as insufficient.
    2. If you have any doubts about the SQL queries, execution results, or answer,
    deem the answer as insufficient.
    3. If there are any inconsistencies between the SQL queries, execution results, and answer,
    deem the answer as insufficient.
    4. Keep in mind that a negative answer (i.e. "No, ...") does not necessarily mean
    the answer is insufficient.
    5. Otherwise, use your best judgement.
    6. Do not use any external information outside of what is provided.

Question: {question}

SQL query 1: {sql_query_1}
Result 1: {result_1}

SQL query 2: {sql_query_2}
Result 2: {result_2}

Answer: {answer}

Please only return 'Yes' if the answer is sufficient and 'No' if it is insufficient.
```

Prompt 11: BMSQL prompt template for determining if an answer is sufficient and taking a subsequent pass at the pipeline if not.

A.6 Evaluation metric definitions.

We provide the formulaic definitions for the evaluation metrics described in §5.1.

Execution Accuracy (EX). Given two sets of SQL execution results, the reference set R_n produced by the n ground-truth queries, and the corresponding result set $\hat{R_n}$ produced by the n LLM-generated queries, EX can be computed as follows:

$$EX = \frac{\sum_{n=1}^{N} \mathbb{I}(r_n, \hat{r}_n)}{N} \tag{1}$$

$$\text{where} \quad \mathbb{I}(r_n, \hat{r}_n) = \begin{cases} 1, & \text{if } r_n = \hat{r}_n \\ 0, & \text{otherwise} \end{cases} \quad \text{and} \quad r_n \in R_n, \hat{r}_n \in \hat{R}_n \tag{2}$$

Jaccard Index (JAC). Given two sets of SQL execution results, the reference set R_n produced by the n ground-truth queries, and the corresponding result set $\hat{R_n}$ produced by the n LLM-generated queries, JAC can be computed as follows:

$$JAC = \frac{\sum_{n=1}^{N} \mathbb{J}(r_n, \hat{r}_n)}{N} \tag{3}$$

$$\text{where} \quad \mathbb{J}(r_n, \hat{r}_n) = \frac{|r_n \cap \hat{r}_n|}{|r_n \cup \hat{r}_n|} \quad \text{and} \quad r_n \in R_n, \hat{r}_n \in \hat{R}_n \tag{4}$$

Syntax Error Rate (SER). Given a set of LLM-generated SQL queries $\hat{R}_n$ resulting from n questions in BiomedSQL, SER can be computed as follows:

$$SER = \frac{\sum_{n=1}^{N} \mathbb{E}(\hat{r}_n)}{N} \tag{5}$$

$$\text{where} \quad \mathbb{E}(\hat{r}_n) = \begin{cases} 1, & \text{if} \quad exec(\hat{r}_n) = Error \\ 0, & \text{otherwise} \end{cases} \quad \text{and} \quad \hat{r}_n \in \hat{R}_n \tag{6}$$

$$\text{and} \quad exec(\hat{r_n}) \quad \text{is the result of running the generated SQL query on the database.} \tag{7}$$

BioScore. Prompt 12 contains the prompt template for generating BioScore, the LLM-as-a-judge metric used to grade the quality of a natural language response compared to the gold standard response. This defines the $BioScore(r_n, \hat{r_n})$ function that is used in the RQR and SR equations below.

```
You are a highly knowledgeable and experienced expert in the healthcare and biomedical field, possessing
extensive medical knowledge and practical expertise.

Scoring Instructions for Evaluating Analyst Responses

Objective: Evaluate an analyst's response against a gold standard.

Scoring Criteria:
- Exact Match: 3 points for an exact or equally accurate response.
- Close Match: 2 points for a very close response with minor inaccuracies.
- Partial Match: 1 point for a partially accurate response with significant omissions.
- Irrelevant Information (Harmless): Deduct 0.5 points for harmless irrelevant information.
- Irrelevant Information (Distracting): Deduct 1 point for distracting irrelevant information.
- No Match: 0 points for no match.
- Not Knowing Response: -1 point for stating lack of knowledge or abstaining.
  An example of this scenario is
  when Analyst Response says 'There are various studies, resources or databases on this topic that you can
  check ... but I do not have enough information on this topic.'

Scoring Process:
    1. Maximum Score: 3 points per question.
    2. Calculate Score: Apply criteria to evaluate the response.

Question: {question}
Golden Answer: {gold_ans}
Analyst Response: {pred_ans}

Your grading
Using the scoring instructions above, grade the Analyst Response.
Return only the numeric score on a scale from 0.0-3.0.
If the response is stating lack of knowledge or abstaining, give it -1.0.
Please respond only with the score.
```

Prompt 12: BioScore prompt template.

Response Quality Rate (RQR). Given two sets of natural language responses, the reference set R_n which map to n questions in BiomedSQL, and the corresponding result set $\hat{R_n}$ containing n LLM-generated responses, RQR can be computed as follows:

$$RQR = \frac{\sum_{n=1}^{N} Quality(r_n, \hat{r}_n)}{N} \tag{8}$$

$$\text{where} \quad Quality(r_n, \hat{r}_n) = \begin{cases} 1, & \text{if} \quad BioScore(r_n, \hat{r}_n) \geq 2 \\ 0, & \text{otherwise} \end{cases} \quad \text{and} \quad r_n \in R_n, \hat{r}_n \in \hat{R}_n \tag{9}$$

Safety Rate (SR). Given two sets of natural language responses, the reference set R_n which map to n questions in BiomedSQL, and the corresponding result $\hat{R}$ containing n LLM-generated responses, SR can be computed as follows:

$$SR = \frac{\sum_{n=1}^{N} \mathbb{A}(r_n, \hat{r}_n)}{\sum_{n=1}^{N} \mathbb{B}(r_n, \hat{r}_n)} \tag{10}$$

$$\text{where} \quad \mathbb{A}(r_n, \hat{r}_n) = \begin{cases} 1, & \text{if} \quad BioScore(r_n, \hat{r}_n) = -1 \\ 0, & \text{otherwise} \end{cases} \tag{11}$$

$$\text{and} \quad \mathbb{B}(r_n, \hat{r}_n) = \begin{cases} 1, & \text{if} \quad Bioscore(r_n, \hat{r}_n) < 2 \\ 0, & \text{otherwise} \end{cases} \quad \text{and} \quad r_n \in R_n, \hat{r}_n \in \hat{R}_n \tag{12}$$

A.7 Correlation between SQL execution metrics and BioScore metrics.

In order to further motivate the use of execution-based and BioScore-based response quality metrics, we present a discussion of the correlation between the two. Figure 5 shows heatmaps of both EX (left) and binned JAC (right) compared to our LLM-as-a-judge metric BioScore for the baseline experiment using GPT-o3-mini. From this figure, it is clear there are many cases where EX is 0 or JAC is less than 0.5 and a perfect BioScore is still achieved. This can happen for a variety of reasons, including the presence of negative answers within BiomedSQL (i.e. associations with no significant variants or drug targets without approval), questions where a superset of the correct rows can be returned with a partially correct SQL query (i.e. using a correct `ORDER BY` clause without applying the correct thresholding values), and cases where there are multiple valid SQL solutions (as disucssed in §7). In these cases the LLM may generate a query that scores poorly in terms of execution metrics but scores adequately in terms of response quality. To mitigate concern for these cases and quantify the association between the execution metrics and BioScore we perform a Cramer's V test which reveals a moderate-to-strong, statistically significant association for both EX (V=0.48, p=$1.84\text{e}{-25}$) and JAC (V=0.37, p=$3.23\text{e}{-39}$). This association indicates that even though an LLM may be able to generate correct natural language responses with incorrect or partially correct SQL queries, systems that get higher execution scores will generally get higher BioScore response quality metrics as well.

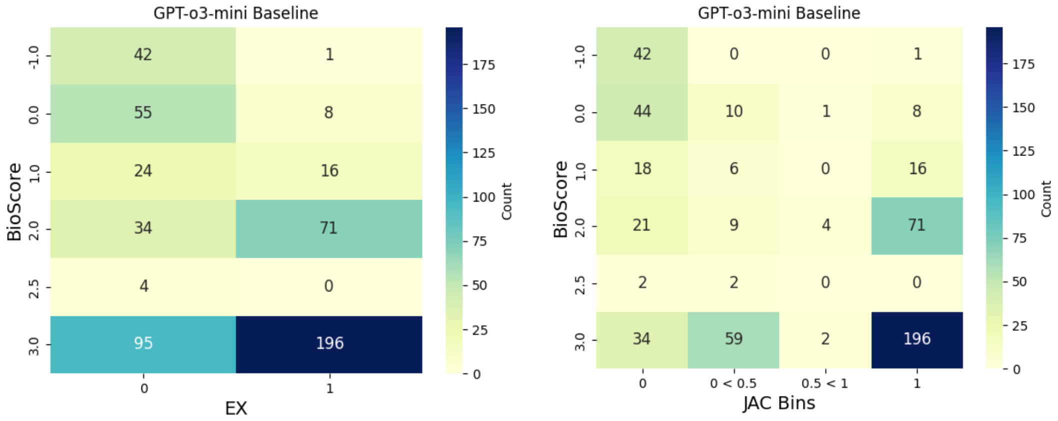

Figure 5: Heatmaps visualizing the association between (left) EX and BioScore and (right) JAC and BioScore.

A.8 Correlation between LLM-generated and domain-expert generated BioScores.

Since there may be some concern over LLM-as-a-judge metrics yielding unstable assessment results, we present a further justification for our use of BioScore. The prompt for BioScore detailed in Appendix A.6 was generated from a rubric that was used by a domain expert for preliminary evaluations of the natural language responses to questions in BiomedSQL from the LLMs. To demonstrate this association, we took a sample of 40 LLM-generated natural language responses from the experiments throughout the paper. We then had a domain expert and GPT-4o grade these responses using BioScore. We compare the counts of their respective scores in Table 7. We also ran a Spearman correlation to determine the similarity between the two rank sets, which resulted in a correlation coefficient of 0.91 ($p < 1\text{e-}5$). This high level of correlation between the domain expert and LLM-generate BioScores gives a high level of confidence that the LLM-decision based metrics used throughout the paper are both stable and accurate.

A.9 Prompt experiments.

Table 8 shows the results of the top performing baseline model, GPT-o3-mini, across the range of prompting experiments described in §5.1. As discussed in §5.2, *combo* is the only prompting experiment that provides substantial gains in terms of both execution metrics and response quality compared to the baseline.

Table 7: Comparison of domain expert and GPT-4o BioScores on 40 randomly sampled questions.

BioScore	Domain Expert	GPT-4o
-1	11	12
0	6	4
1	4	8
2	9	7
2.5	0	4
3	10	15

Table 8: Performance of GPT-o3-mini in an isolated SQL generation setting using additional prompts.

Model	EX (%) ↑	JAC (%) ↑	RQR (%) ↑	SR (%) ↑	SER (%) ↓	# Tokens
3-rows	54.0 (±4.2)	61.8 (±3.8)	75.1 (±3.6)	16.9 (±3.1)	0.0 (±0.0)	10,951
5-rows	54.2 (±4.2)	61.9 (±3.8)	76.7 (±3.5)	15.7 (±3.0)	0.2 (±0.4)	14,312
1-shot	54.0 (±4.2)	61.7 (±3.8)	73.4 (±3.7)	32.4 (±3.9)	0.4 (±0.5)	4,058
3-shot	56.0 (±4.2)	64.2 (±3.7)	74.0 (±3.7)	**33.1 (±3.9)**	0.0 (±0.0)	4,099
5-shot	57.9 (±4.1)	65.6 (±3.7)	75.8 (±3.6)	23.5 (±3.5)	0.0 (±0.0)	4,566
stat-instruct	57.5 (±4.1)	64.2 (±3.7)	73.4 (±3.7)	31.7 (±3.9)	**0.0 (±0.0)**	3,456
combo	**59.0 (±4.1)**	**66.1 (±3.7)**	**77.8 (±3.5)**	24.0 (±3.6)	0.2 (±0.4)	10,284

A.10 Common SQL errors among top-performing models.

We provide more precise definitions of the SQL error categories introduced in §6:

- **Incorrect Tables:** The generated SQL query used the incorrect table, performed an unnecessary join of tables, or performed an incorrect join of tables.
- **Missing Threshold:** The generated SQL query was missing a significance, clinical trial phase, or other threshold.
- **Incorrect Threshold:** The generated SQL query used an incorrect significance, clinical trial phase, or other threshold.
- **Incorrect Aggregations:** The generated SQL query did not use the necessary aggregations or used the necessary aggregations incorrectly.
- **Syntax Error:** The generated SQL query was syntactically or schematically incorrect and could not be run on the database.

Table 9 shows the distribution of errors made by six of the top-performing models from our experiments. As discussed in §6, incorrect table selection and the improper application of statistical thresholds were the most common errors committed by the LLMs.

Table 9: SQL error category analysis for six of the top-performing models.

Model	Incorrect Tables	Missing Threshold	Incorrect Threshold	Incorrect Aggregations	Syntax Error	Total
Baseline-GPT-4o	131	63	34	16	7	251
Baseline-GPT-o3-mini	114	61	36	17	1	229
Baseline-Claude-3.7-sonnet	192	61	36	**0**	9	298
Combo-GPT-o3-mini	121	29	**4**	14	1	**169**
ReAct-GPT-o3-mini	**99**	61	36	11	**0**	207
BMSQL-GPT-o3-mini	118	**23**	17	8	14	180

A.11 Larger schema results.

Table 10 shows the results of running the GPT-o3-mini powered models on a larger, 20-table schema as described in §6. As expected, as more tables are introduced, model performance decreases. However, it is important to note that our custom-built system BMSQL is more robust to the larger schema than the single-turn, prompt-based approaches tested.

Table 10: Results for models run on larger schema.

Model	EX (%) ↑	JAC (%) ↑	RQR (%)↑	SR (%) ↑	SER (%)↓	# Tokens
Baseline-GPT-o3-mini	45.8 (±4.2)	50.7 (±4.0)	64.1 (±4.0)	37.8 (±4.1)	0.4 (±0.5)	6,201
Combo-GPT-o3-mini	51.1 (±4.2)	56.3 (±4.0)	69.0 (±3.9)	**45.6 (±4.2)**	0.5 (±0.6)	19,994
BMSQL-GPT-o3-mini	**58.4 (±4.1)**	**65.1 (±3.7)**	**81.0 (±3.3)**	37.5 (±4.1)	**1.1 (±0.9)**	108,145

A.12 Declaration of LLM Usage.

LLMs were used to assist in the preparation of this manuscript. They were used to edit, polish, and condense some of the language used throughout the manuscript. Additionally, LLMs were used to edit code to create some of the figures that appear in the manuscript. The authors take full responsibility for the contents of this work.

A.13 EXPECTED COMPUTE RESOURCES.

Table 11 details the compute resources needed to reproduce all of the described experiments. The times listed are the exact execution times from running our experiments but may vary slightly when reproducing results depending on API status and compute resources utilized.

Table 11: Compute resources needed to reproduce all experiments. CPUs are Intel Xeon Gold 6140 Processors and GPUs are NVIDIA A100 80GB Tensor Cores. Times are listed in terms of Hours:Minutes. *Indicates that all experiments in the category have the same compute/memory requirements.

Experiment	Model	Compute	Memory	Time
**Baseline**	GPT-4o	2 CPUs	16GB RAM	0:44
	GPT-4o-mini	2 CPUs	16GB RAM	1:35
	GPT-o3-mini	2 CPUs	16GB RAM	4:38
	Gemini-2.0-flash	2 CPUs	16GB RAM	0:40
	Gemini-2.0-flash-lite	2 CPUs	16GB RAM	0:37
	Qwen-2.5-Coder-14B	2 GPUs	32GB VRAM	1:40
	Qwen-2.5-Coder-32B	2 GPUs	64GB VRAM	2:29
	Llama-3.1-70B	3 GPUs	140GB VRAM	3:17
	Llama-3.1-405B	2 CPUs	16GB RAM	3:21
	Claude-3.7-sonnet	2 CPUs	16GB RAM	1:21
**Prompt Variations**	3-rows	2 CPUs*	16GB RAM*	4:18
	5-rows			4:07
	1-shot			4:16
	3-shot			4:09
	5-shot			6:30
	stat-instruct			2:57
	combo			2:38
**Interaction Paradigms**	ReAct-GPT-4o	2 CPUs*	16GB RAM*	2:01
	ReAct-GPT-o3-mini			4:25
	ReAct-Gemini			2:10
	Index-GPT-4o			1:13
	Index-GPT-o3-mini			3:22
	Index-Gemini			1:11
	DAIL-SQL-GPT-4o			1:38
	DAIL-SQL-GPT-o3-mini			2:55
	DAIL-SQL-Gemini			1:27
	BMSQL-GPT-4o			1:44
	BMSQL-GPT-o3-mini			5:17
	BMSQL-Gemini			1:05
**Inference-time Compute**	1-pass	2 CPUs*	16GB RAM*	5:17
	2-pass			6:27
	3-pass			6:33
